# Supplementary material for: A Humanized Yeast Phenomic Model of Deoxycytidine Kinase to Predict Genetic Buffering of Nucleoside Analog Cytotoxicity
Source: Genes (Basel). 2019 Sep 30;10(10):770. doi: 10.3390/genes10100770 (PMC6826991; doi:10.3390/genes10100770)
Supplement: Supplementary file 1 [file genes-10-00770-s001.zip › Additional_Files/Additional_File7_GOTermHeatmaps.zip!/Additional_File7_GOTermHeatmaps/tRNA_aminoacylation.pdf]

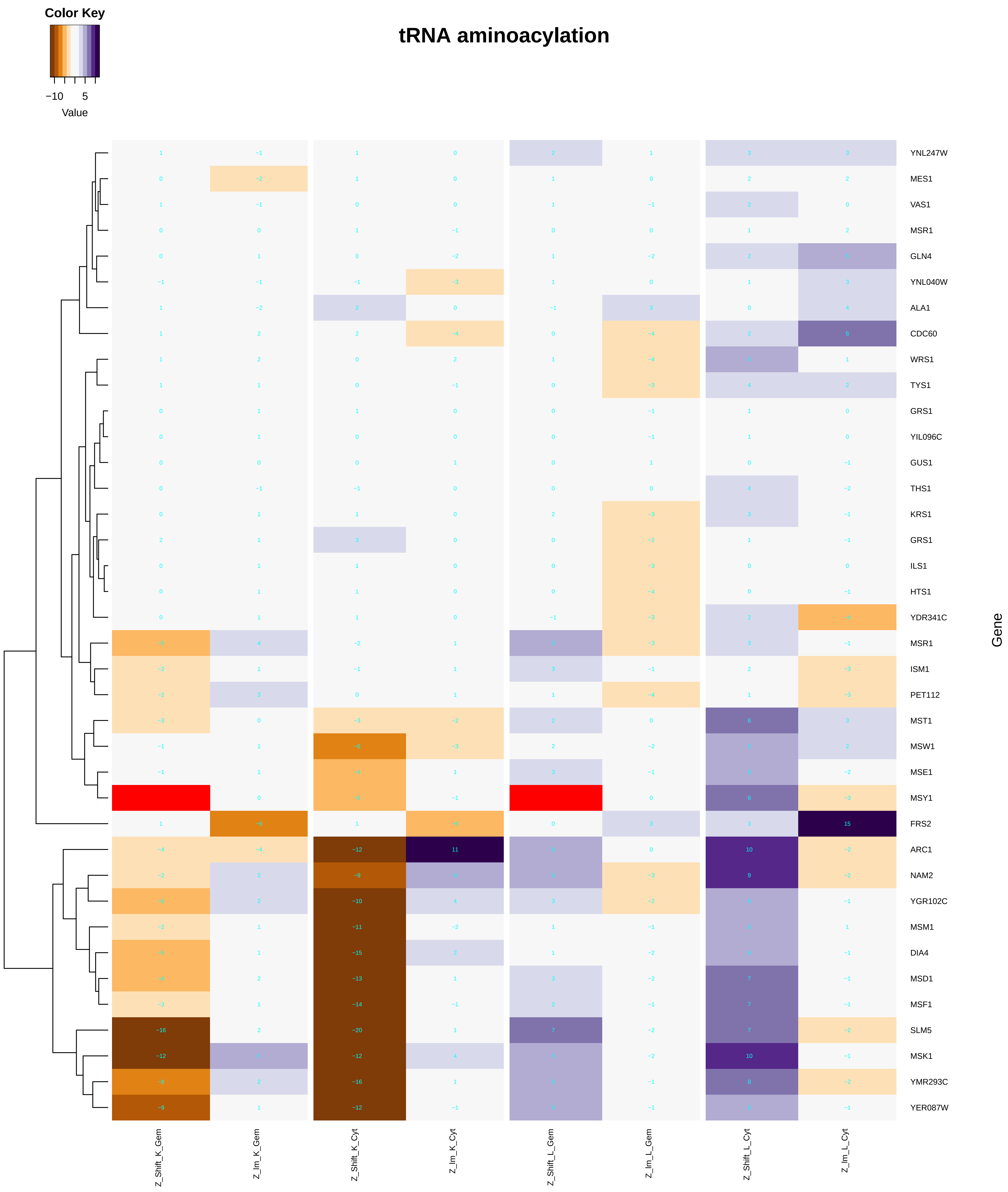
tRNA aminoacylation
Color Key
−10
5
Value
YNL247W
MES1
VAS1
MSR1
GLN4
YNL040W
ALA1
CDC60
WRS1
TYS1
GRS1
YIL096C
GUS1
THS1
KRS1
GRS1
ILS1
HTS1
YDR341C
MSR1
ISM1
PET112
MST1
MSW1
MSE1
MSY1
FRS2
ARC1
NAM2
YGR102C
MSM1
DIA4
MSD1
MSF1
SLM5
MSK1
YMR293C
YER087W
Gene
Z_Shift_K_Gem
Z_Im_K_Gem
Z_Shift_K_Cyt
Z_Im_K_Cyt
Z_Shift_L_Gem
Z_Im_L_Gem
Z_Shift_L_Cyt
Z_Im_L_Cyt

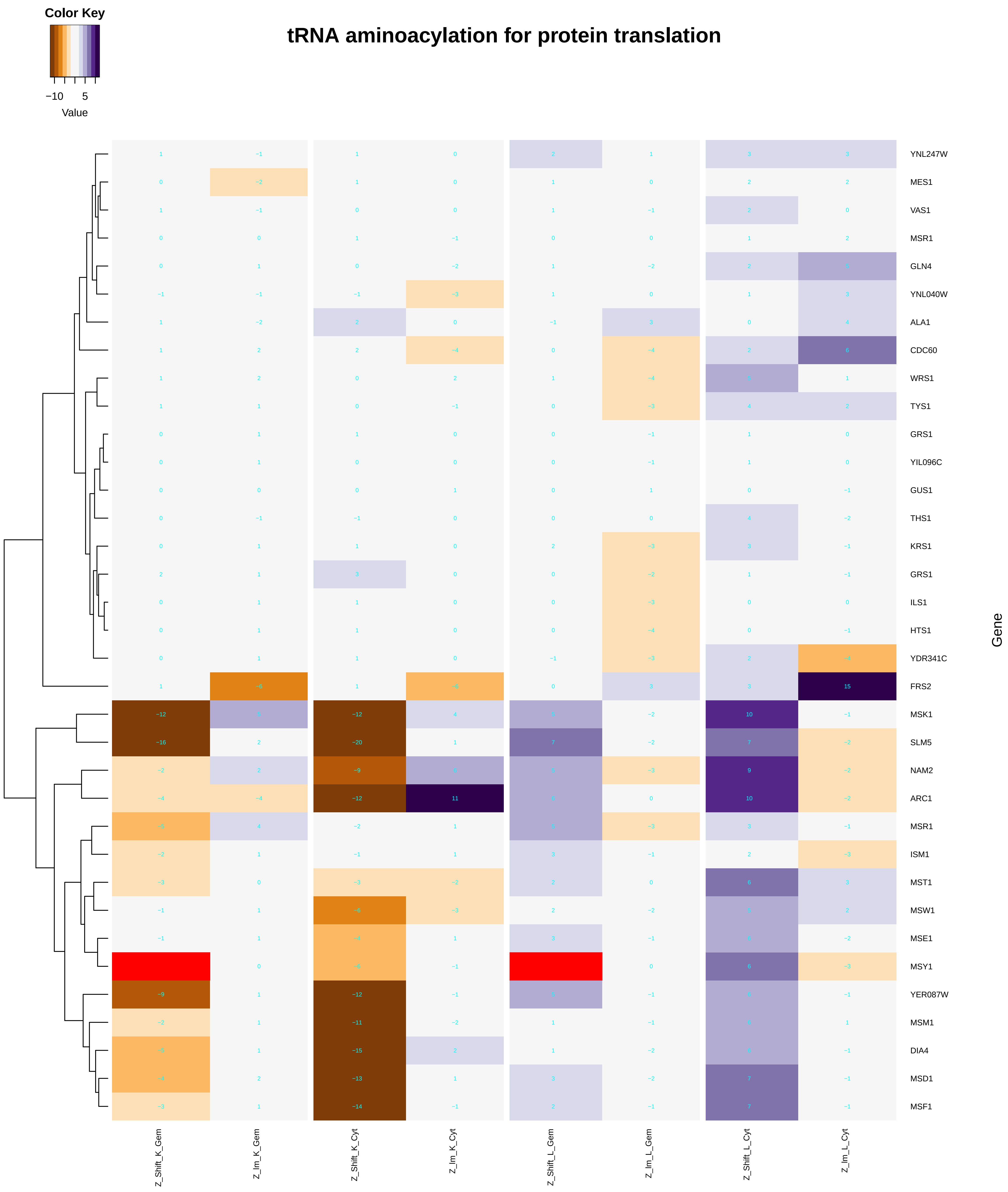
tRNA aminoacylation for protein translation
Color Key
−10
5
Value
YNL247W
MES1
VAS1
MSR1
GLN4
YNL040W
ALA1
CDC60
WRS1
TYS1
GRS1
YIL096C
GUS1
THS1
KRS1
GRS1
ILS1
HTS1
YDR341C
FRS2
MSK1
SLM5
NAM2
ARC1
MSR1
ISM1
MST1
MSW1
MSE1
MSY1
YER087W
MSM1
DIA4
MSD1
MSF1
Gene
Z_Shift_K_Gem
Z_Im_K_Gem
Z_Shift_K_Cyt
Z_Im_K_Cyt
Z_Shift_L_Gem
Z_Im_L_Gem
Z_Shift_L_Cyt
Z_Im_L_Cyt

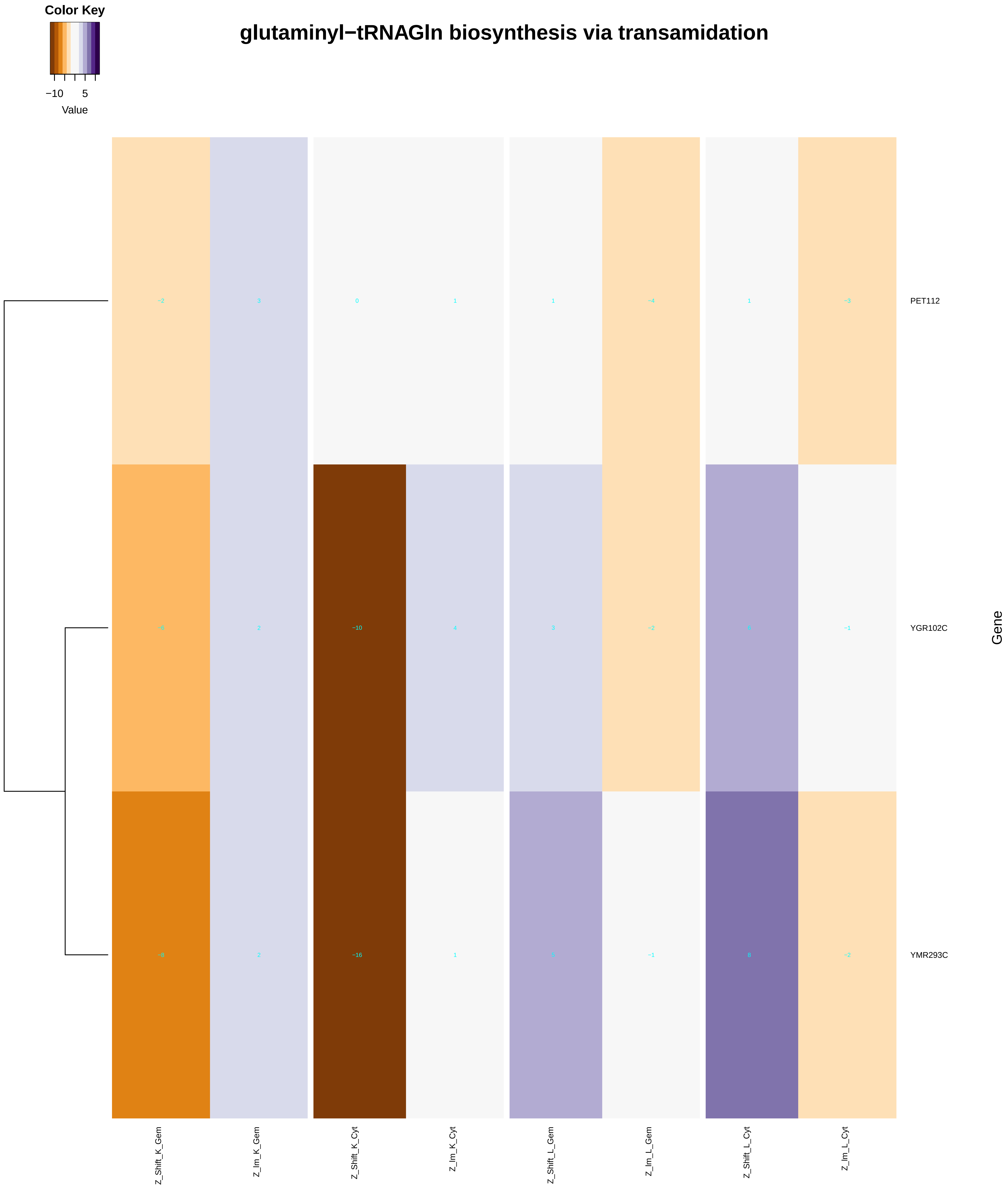
Color Key
−10
5
Value
glutaminyl−tRNAGln biosynthesis via transamidation
−2
3
0
1
1
−4
1
−3
PET112
−6
2
−10
4
3
−2
6
−1
YGR102C
−8
2
−16
1
5
−1
8
−2
YMR293C
Gene
Z_Shift_K_Gem
Z_Im_K_Gem
Z_Shift_K_Cyt
Z_Im_K_Cyt
Z_Shift_L_Gem
Z_Im_L_Gem
Z_Shift_L_Cyt
Z_Im_L_Cyt

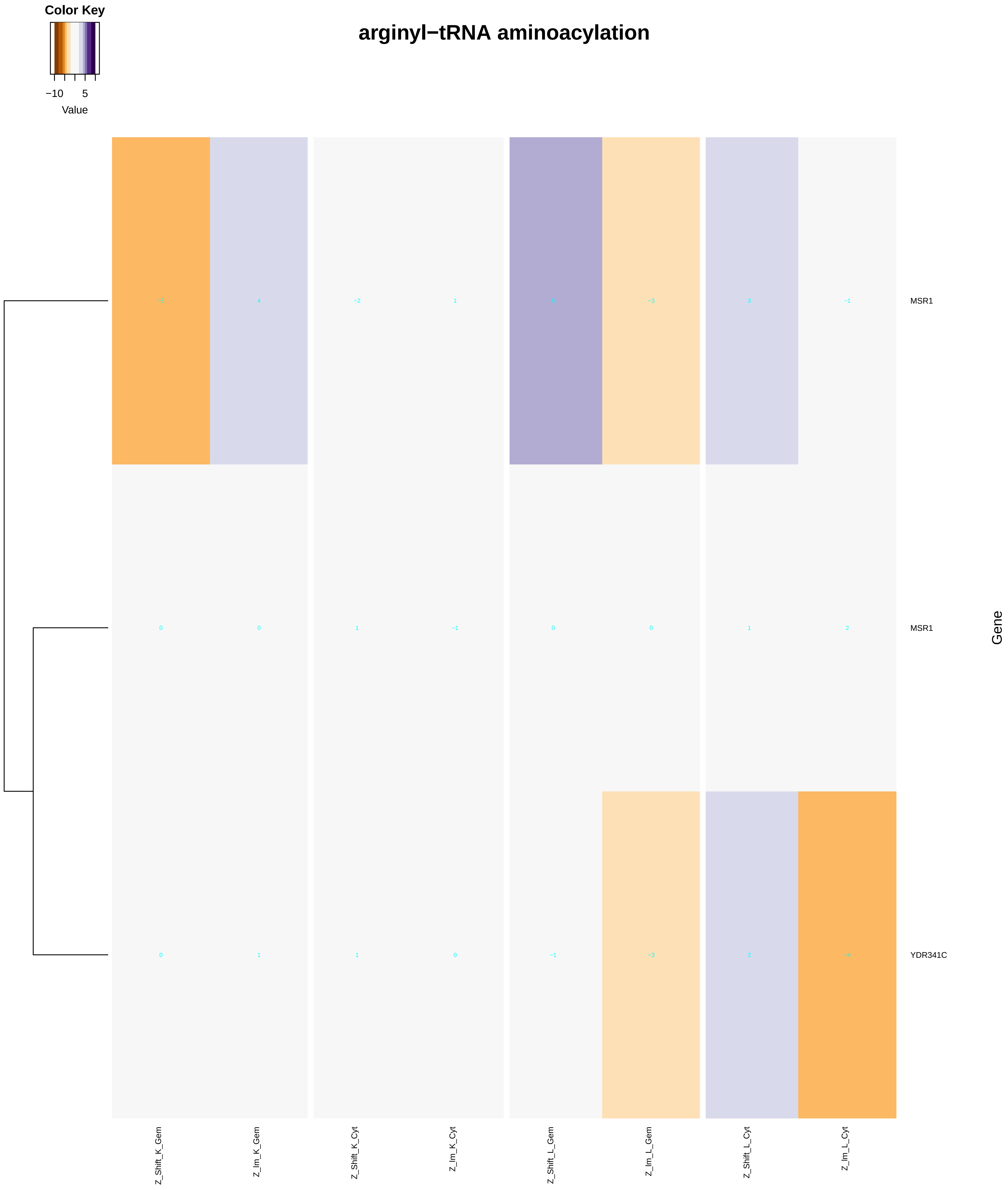

Color Key
−10
5
Value
arginyl−tRNA aminoacylation
MSR1
MSR1
YDR341C
Gene
Z_Shift_K_Gem
Z_Im_K_Gem
Z_Shift_K_Cyt
Z_Im_K_Cyt
Z_Shift_L_Gem
Z_Im_L_Gem
Z_Shift_L_Cyt
Z_Im_L_Cyt

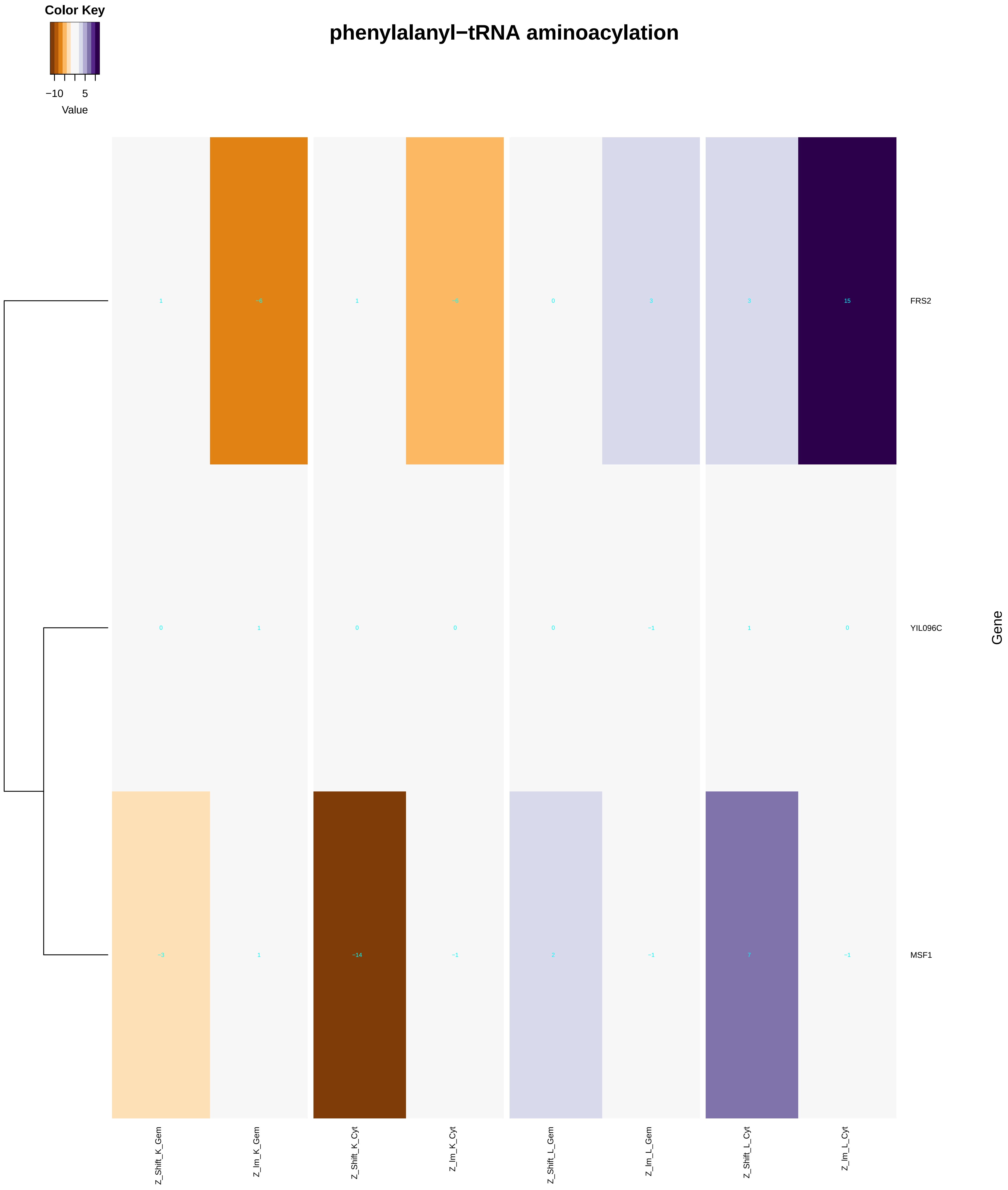
Color Key
−10
5
Value
phenylalanyl−tRNA aminoacylation
1
−6
1
−6
0
3
3
15
FRS2
0
1
0
0
0
−1
1
0
YIL096C
−3
1
−14
−1
2
−1
7
−1
MSF1
Gene
Z_Shift_K_Gem
Z_Im_K_Gem
Z_Shift_K_Cyt
Z_Im_K_Cyt
Z_Shift_L_Gem
Z_Im_L_Gem
Z_Shift_L_Cyt
Z_Im_L_Cyt

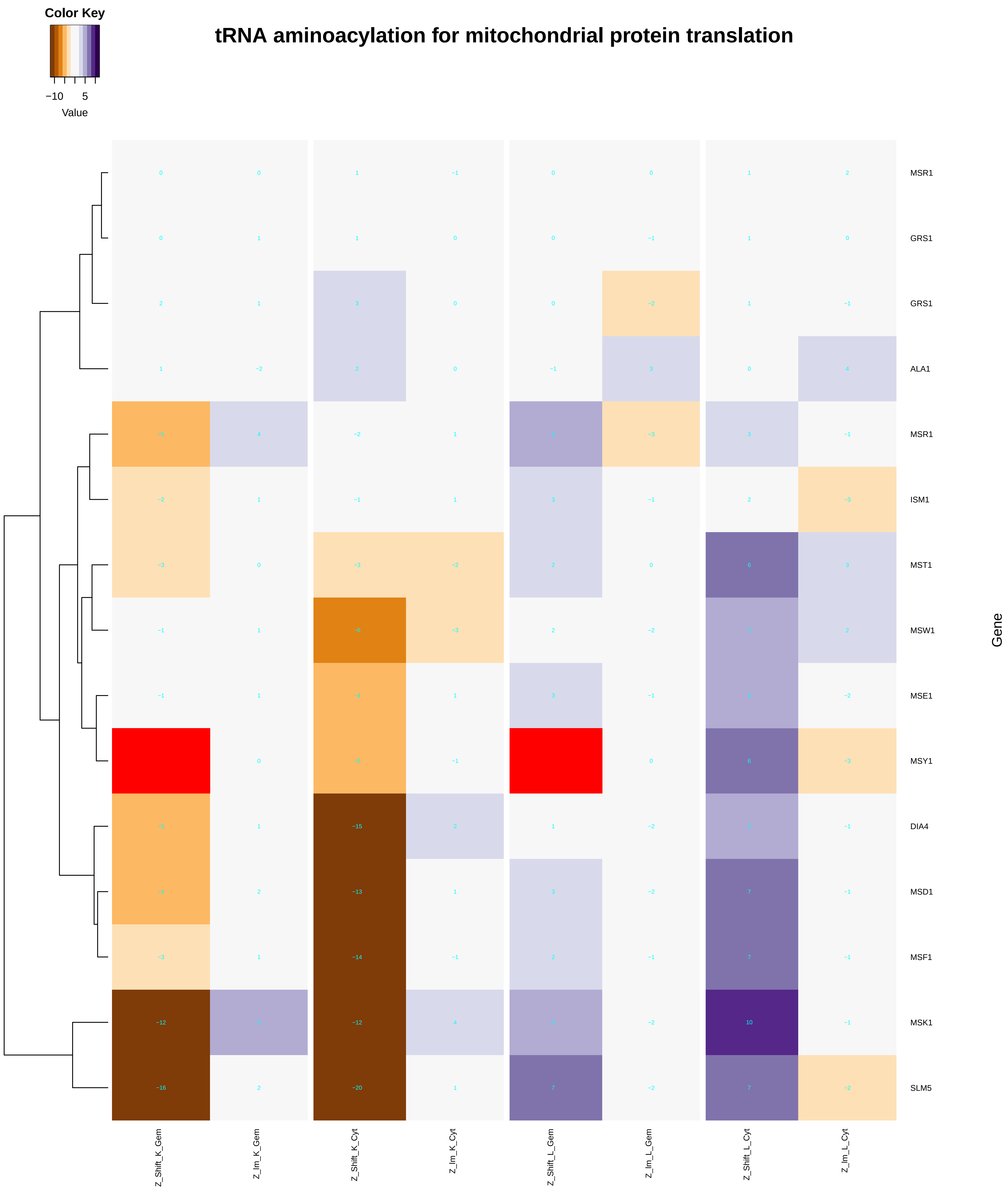

tRNA aminoacylation for mitochondrial protein translation
Color Key
−10
5
Value
MSR1
GRS1
GRS1
ALA1
MSR1
ISM1
MST1
MSW1
MSE1
MSY1
DIA4
MSD1
MSF1
MSK1
SLM5
Gene
Z_Shift_K_Gem
Z_Im_K_Gem
Z_Shift_K_Cyt
Z_Im_K_Cyt
Z_Shift_L_Gem
Z_Im_L_Gem
Z_Shift_L_Cyt
Z_Im_L_Cyt
